# Transcriptional Profiling Uncovers a Network of Cholesterol-Responsive Atherosclerosis Target Genes

**DOI:** 10.1371/journal.pgen.1000036

**Published:** 2008-03-14

**Authors:** Josefin Skogsberg, Jesper Lundström, Alexander Kovacs, Roland Nilsson, Peri Noori, Shohreh Maleki, Marina Köhler, Anders Hamsten, Jesper Tegnér, Johan Björkegren

**Affiliations:** 1 The Computational Medicine Group, Karolinska Institutet, Karolinska University Hospital Solna, Stockholm, Sweden; 2 Atherosclerosis Research Unit, Center for Molecular Medicine, Department of Medicine, Karolinska Institutet, Karolinska University Hospital Solna, Stockholm, Sweden; 3 Division of Computational Biology, Department of Physics, Linköpings Institute for Technology, Linköping University, Linköping, Sweden; The Jackson Laboratory, United States of America

## Abstract

Despite the well-documented effects of plasma lipid lowering regimes halting atherosclerosis lesion development and reducing morbidity and mortality of coronary artery disease and stroke, the transcriptional response in the atherosclerotic lesion mediating these beneficial effects has not yet been carefully investigated. We performed transcriptional profiling at 10-week intervals in atherosclerosis-prone mice with human-like hypercholesterolemia and a genetic switch to lower plasma lipoproteins (*Ldlr* ^−/−^ *Apo* ^100/100^ *Mttp* ^flox/flox^ Mx1-*Cre*). Atherosclerotic lesions progressed slowly at first, then expanded rapidly, and plateaued after advanced lesions formed. Analysis of lesion expression profiles indicated that accumulation of lipid-poor macrophages reached a point that led to the rapid expansion phase with accelerated foam-cell formation and inflammation, an interpretation supported by lesion histology. Genetic lowering of plasma cholesterol (e.g., lipoproteins) at this point all together prevented the formation of advanced plaques and parallel transcriptional profiling of the atherosclerotic arterial wall identified 37 cholesterol-responsive genes mediating this effect. Validation by siRNA-inhibition in macrophages incubated with acetylated-LDL revealed a network of eight cholesterol-responsive atherosclerosis genes regulating cholesterol-ester accumulation. Taken together, we have identified a network of atherosclerosis genes that in response to plasma cholesterol-lowering prevents the formation of advanced plaques. This network should be of interest for the development of novel atherosclerosis therapies.

## Author Summary

Atherosclerosis is present in the major arteries of all adults. In industrial societies, atherosclerosis progression in ∼50% of adults leads to clinical manifestations such as stroke and myocardial infarction, and eventually death. Lowering circulating LDL-cholesterol levels can slow atherosclerosis progression and even cause regression. Yet, little is known about the genes in the atherosclerotic arterial wall that mediate those effects. To identify such genes, we studied genetically modified mice in which high levels of human-like LDL-cholesterol cause rapid progression of atherosclerosis; the mice also had a genetic “switch” to lower LDL-cholesterol. Lowering LDL-cholesterol at a critical point before advanced plaques developed stopped lesion progression. Analysis of gene expression in response to the lowering of plasma LDL-cholesterol revealed 37 lesion genes as possible mediators of this effect. We validated some of these genes in macrophages using siRNA incubated with acetylated-LDL to mimic foam cells, which are central to atherosclerosis progression. “Reverse engineering” of whole-genome expression data from these experiments revealed a regulatory gene network of cholesterol-responsive atherosclerosis genes that control foam cell formation. This network and the individual genes within it merit further attention as targets for drugs to prevent the transformation of early harmless lesions into advanced, clinically significant plaques.

## Introduction

Atherosclerosis is a lifelong, progressive disease that becomes clinically significant in 50% of the population, leading to myocardial infarction (MI), stroke, and eventually death. Statin-based lipid-lowering regimens reduce morbidity and mortality from both MI and stroke [1]. Aggressive statin regimens can even cause regression of atherosclerosis [2] but sometimes generate severe side effects. To fully exploit the beneficial effects of lipid lowering, we need a better understanding of the transcriptional changes induced by lowering plasma lipoproteins [3]. Whole-genome measurement technologies developed in the aftermath of the human [4],[5] and mouse [6] genome projects offer the opportunity to uncover entire repertoires of changes in gene expression related to complex diseases like atherosclerosis and reveal their interplay in regulatory networks. We used the *Ldlr* ^−/−^ *Apo* ^100/100^ *Mttp* ^flox/flox^Mx1-*Cre* mouse model [7] that has a plasma lipoprotein profile similar to that of familial hypercholesterolemia (*Ldlr* ^−/−^ *Apob* ^100/100^) and a genetic switch to block hepatic synthesis of lipoproteins and thereby lower plasma lipoproteins (*Mttp* ^flox/flox^Mx1-*Cre*) to investigate the effects of plasma cholesterol-lowering on atherosclerosis development and to identify cholesterol-responsive atherosclerosis genes.

## Results

### Basic Characteristic and Atherosclerotic Lesion Development

**Figure 1:**
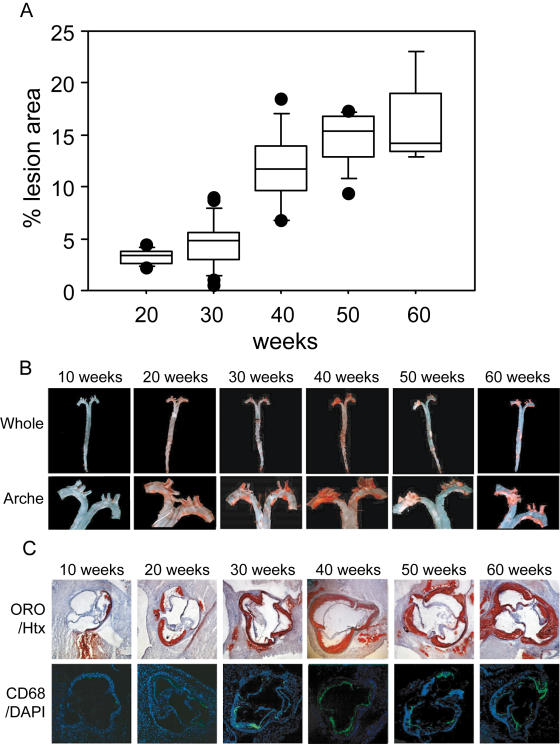
Atherosclerosis progression in *Ldlr* ^−/−^ *Apob^100/100^ Mttp* ^flox/flox^ mice. (A) Box plots of atherosclerosis progression at 20 (n = 12), 30 (n = 25), 40 (n = 15), 50 (n = 15), and 60 weeks (n = 10). *P*<0.05, 20 *vs.* 30 weeks; *P*<0.0001, 30 *vs.* 40 weeks; *P*<0.02, 40 *vs.* 50 weeks. Values are surface lesion areas assessed by Sudan IV staining of pinned-out aortas and given as a percentage of the surface of the entire aorta. Boxes enclose values between the 75^th^ and 25^th^ percentiles, bars indicate values between the 90^th^ and 10^th^ percentiles, and black dots indicate individual observations outside these boundaries. (B) Representative aortic trees (above) and arches (below). (C) Representative sections of the aortic root were stained with Oil-red-O and counterstained with hematoxylin (Htx) or show CD68 fluorescence and counterstained with DAPI.

The mice were sacrificed at 10, 20, 30, 40, 50 and 60 weeks of age. Lesion area and morphological changes were assessed in a total of 87 *Ldlr* ^−/−^ *Apob^100/100^Mttp* ^flox/flox^Mx1-*Cre* mice (Figure 1A–1C). Only occasional spots of Sudan IV staining were detected at 10 weeks (n = 10, not shown), but lesions were detected in all mice at 20 weeks. Lesion size increased by ∼1.6% between weeks 20 and 30 (*P* = 0.05) and by ∼7.2% between weeks 30 and 40 (*P*<0.0001). At 20 weeks, the main morphological feature was fatty streaks (red transparent areas with diffuse boundaries); no plaques were detected. At 30 weeks, all mice had small plaques (red nontransparent areas with distinct boundaries) in the aortic arch that had expanded markedly at 40 weeks. Thereafter plaque growth was restrained (Figure 1A and 1B). The area of Oil-red-O staining in the aortic root followed a similar progression curve as the Sudan IV lesion staining whereas CD68 staining increased sharply between week 20 and 30 (Figure 1C). Plasma cholesterol increased slightly with age whereas plasma triglyceride and glucose levels were unchanged (Table 1).

**Table 1:** Plasma cholesterol, triglyceride, and glucose concentrations of representative *Ldlr* ^−/−^ *Apob^100/100^Mttp* ^flox/flox^Mx1-*Cre* mice.

Plasma level (mg/dl)	Age (weeks)					
	10.6±0.4 (n = 7)	21.3±1.1 (n = 5)	29.6±0.0 (n = 6)	40.3±0.0 (n = 5)	50.5±0.8 (n = 5)	61.0±0.0 (n = 4)
Cholesterol	361.2±64.2	345.7±65.3	427.0±72.4	461.9±14.1	427.2±74.2	527.3±71.5
Triglycerides	83.0±25.8	84.1±10.5	87.8±17.0	84.7±20.0	108.6±26.2	89.6±38.3
Glucose	359.6±50.6	425.4±76.2	369.8±37.8	311.4±28.5	350.6±19.6	376.0±38.3

Values are mean ±SD. There were no statistically significant differences between time points (*P*>0.05).

### Transcriptional Profiling of Lesion Development

Next, we identified transcriptional changes of atherosclerosis development using 32 cDNA arrays (5 to 7 per time point). Of 19,879 genes (Mouse Genome Informatics Database, www.jax.org), 1259 (6.3%) were differentially expressed according to empirical Bayes statistics [8] at one or more time comparisons (FDR<0.05, n = 1259). Of these, 329 (27%) had previously been related to atherosclerosis (Table S1). Of genes with established roles in atherosclerosis (Table S6), 78% (88/111) were among the differentially expressed genes.

**Figure 2:**
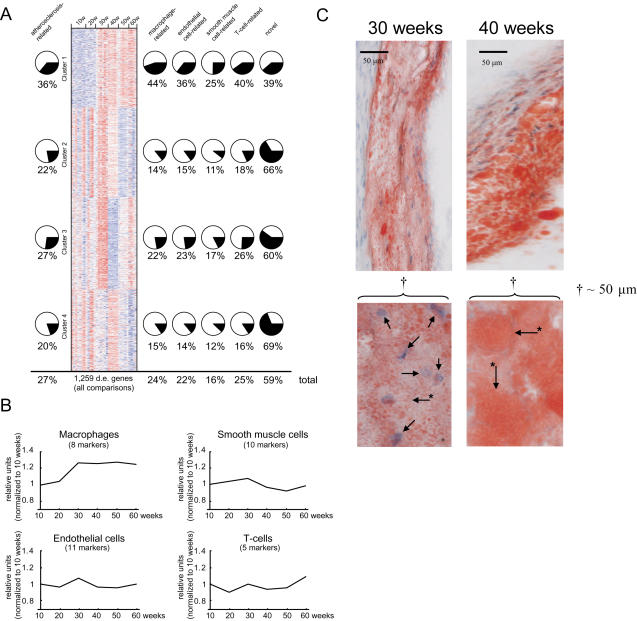
Transcriptional changes of atherosclerosis development. (A) Heat map of clustered mRNA levels (rows; red, high levels; blue, low levels) for genes differentially expressed (d.e.) in at least one pair-wise time-point comparison (FDR<0.05, n = 1259). Each column represents mRNA levels in one mouse (n = 5 to 7 per time point) at 10 to 60 weeks of age. Four gene clusters were identified by the cluster algorithm. Pie charts show percentages of genes related to atherosclerosis (Table S1) on the left and to the four atherosclerosis cell types (Tables S2, S3, S4, and S5) on the right. A substantial number of genes were related to more than one atherosclerosis cell type (Figure S1). The percentage of novel genes (i.e., not related to atherosclerosis or atherosclerosis cell types) in each cluster is shown on the far right. Overall percentages (total) are shown below. (B) Relative expression levels of cell-specific markers of atherosclerosis cell types (Table S7). The number of markers per cell type is indicated. The only statistically significant increase was in the number of foam cells, which increased by ∼30% between 20 and 30 weeks (*P*<0.001) and remained elevated at 60 weeks. (C) Representative sections of the aortic root (top) were stained with Oil-red-O (red in the figure) and counterstained with hematoxylin (Htx) (blue spots in the figure), bar in figure indicate 50 µm; higher-power views of 50 µm showing foam cells at 30 (arrows indicate macrophage Htx-stained nuclei, asterisk marked arrows point to the outer cell membrane) and 40 weeks (asterisk marked arrows point to the outer cell membrane of Oil-red-O-stained fat within macrophages, the nuclei are not visible) are shown below.

Cluster analysis of mRNA levels of differentially expressed genes (n = 1259) generated four distinct clusters (Figure 2A, Table S21). Cluster 1 genes (n = 293, Table S8) were activated during rapid lesion expansion between 30 and 40 weeks (Figure 1A) and remained activated at 60 weeks (Figure 2A). This cluster had the highest percentage (36%) of genes previously related to atherosclerosis or atherosclerosis cell types (Figure 2A, Table S1), and 89% of the genes were related to inflammatory cells (Tables S13, S17, S22).

Gene activities peaked at week 30 in cluster 2 (n = 331; Table S9) and at week 40 in cluster 4 (n = 300; Table S11) and were suppressed at late stages of progression (Figure 2A); 73% had no previous relation to atherosclerosis or atherosclerosis cell types (Tables S14, S16, S18, S20, S23, and S25).

Cluster 3 (n = 339; Table S10) was particularly interesting because the mRNA levels of these genes peaked at 30 weeks and were suppressed at 40 weeks (Figure 2A), coinciding with the rapid lesion expansion phase. This cluster had fewer atherosclerosis-related genes than cluster 1 but more than clusters 2 or 4 and consisted mainly of genes related to carboxylic and lipid metabolism (Tables S15, S19, and S24). Thirteen of 19 TFs are well-established in lipid and energy metabolism, including the peroxisome proliferator activator receptors (PPARs; Ppara (Entrez Gene ID 19013), Ppard (Entrez Gene ID 19015), and Pparg (Entrez Gene ID 19016)) and sterol regulatory element binding factor 2 (Srebf2, Entrez Gene ID 20788), which also have been implicated in the regulation of foam-cell formation.

Cell-type-specific markers (Table S7) were used to investigate changes to the relative cell type contents of the lesion over time. mRNA levels of these markers on the GeneChips indicated that most of the major lesion cell types (smooth muscle and endothelial and T-cells) were relatively stable during lesion progression (Figure 2B). In contrast, macrophages/foam cells increased in number between 20 and 30 weeks and remained at this level until 60 weeks (Figure 2B). This change in lesion macrophage number confirmed the notion obtained from the CD68 staining (Figure 1C).

In summary, the gene expression data suggest that macrophages gradually accumulate in the early phases of lesion development, reaching a density at 30 weeks (Figure 2B), inducing an inflammatory reaction (Figure 2A, cluster 1), increasing lipid accumulation in foam cells (Figure 2A, cluster 3), and causing rapid lesion expansion (Figure 1A and 1B). The lipid accumulation in foam cells was sustained until 40 weeks (Figure 2A, cluster 3). The inflammation persisted in the later phases (Figure 2A, cluster 1).

This interpretation of the expression data was supported by a immunohistochemical examination showing that lipid-poor macrophages had accumulated at 30 weeks and then had expanded and become lipid-rich at 40 weeks (Figure 2C).

### Identification of Plasma Cholesterol-Responsive Atherosclerosis Genes

**Figure 3:**
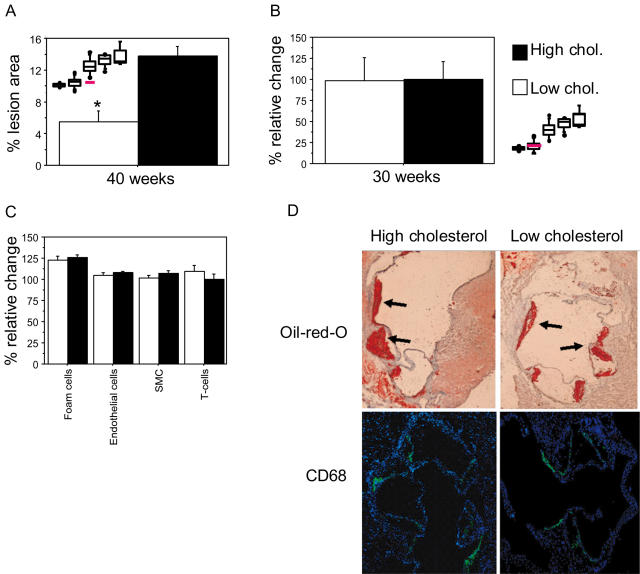
Effect of plasma-cholesterol lowering on lesion progression. Lesion surface area was determined as the percentage of lesion area in relation to the total area of pinned-out aortas from the bifurcation to the aortic root. At 28 weeks of age, mice received intraperitoneal injections of pI-pC to induce recombination of *Mttp* in the liver and were sacrificed 12 weeks later or 1 week after cholesterol lowering had been achieved. High-cholesterol control mice were injected with PBS. (A) At 40 weeks of age, lesion surface area in mice with low plasma cholesterol (i.e., pI-pC-treated, n = 7) had not progressed and differed significantly from that in high-cholesterol controls at 40 weeks (i.e., PBS-treated, n = 6) (*P<0.005). Figure 1A is shown for comparison; red line indicates mice with low plasma cholesterol. (B–D) One week of low levels of cholesterol (30-week-old mice) did not affect lesion size (*P* = 0.96), (B) Shown are percent relative changes in lesion area. Figure 1A is shown for comparison; red line indicates the low-cholesterol group. (C) The numbers of foam cells (*P* = 0.52), endothelial cells (*P* = 0.49), smooth muscle cells (*P* = 0.18) (SMC), and T cells (*P* = 0.34) and (D) the staining of Oil-red-O (upper panels) and fluorescence of anti-rat antibodies binding to rat anti mouse CD68 (lower panels) in representative sections isolated from the aortic root of high-cholesterol (left panels) and low-cholesterol (right panels) mice. Arrows indicate Oil-red-O stained lesions.

To investigate the role of plasma cholesterol in causing the rapid lesion expansion between week 30 and 40, we genetically lowered plasma LDL cholesterol by inducing recombination of *Mttp* (*Ldlr* ^−/−^ *Apob^100/100^Mttp* ^Δ/Δ^) in 30-week-old mice. Plasma cholesterol was reduced >80% (427 to 54±31 mg/L, n = 6) and remained at this level for 10 weeks until sacrifice. At sacrifice, lesion size had not increased and was significantly less than in controls with high cholesterol (Figure 3A). Lesion histology was indistinguishable from that in 30-week-old mice with high plasma cholesterol (not shown). Thus, lowering plasma cholesterol at 30 weeks eliminated the formation of advanced plaques observed in mice with sustained high levels of plasma cholesterol.

To investigate the molecular mechanisms in the atherosclerotic arterial wall induced by the genetic lowering of plasma cholesterol preventing the formation of advanced lesion, we performed additional gene expression profiling of lesion mRNA isolated from mice sacrificed immediately after plasma cholesterol lowering by Cre-mediated recombination of *Mttp* in the liver (Text S1). The mRNA levels of thirty-seven atherosclerosis genes responded strongly to the cholesterol lowering (Table S12). To determine if these changes were true changes in cellular mRNA levels or a consequence of changes in lesion size or the relative contributions of cell types, we examined lesion size and the relative numbers of the four major atherosclerosis cell types. Neither lesion size (Figure 3B) nor cell type distribution (Figure 3C) was affected by the subacute lowering of plasma cholesterol, as judged from cell-surface markers (Table S7), Oil-red-O staining, and immunohistochemical analysis of CD68 expression (Figure 3D) suggesting that the observed changes in mRNA levels were true gene activity changes in response to the plasma cholesterol lowering.

Although the recombination of *Mttp* in the *Ldlr* ^−/−^ *Apob^100/100^Mttp* ^flox/flox^Mx1-*Cre* mouse leading to lowering of plasma cholesterol mainly takes place in the liver, the intraperitoneal pI─pC treatment and the following activation of Cre-recombinase may theoretically in themselves affect gene expression in the atherosclerotic arterial wall. To investigate this possibility, we bred control mice lacking the floxed *Mttp* allele (*Ldlr* ^−/−^ *Apob^100/100^Mttp* ^wt/wt^Mx1-*Cre*) and treated these mice with pI-pC (n = 4) and PBS (n = 5). As expected, the pI-pC treatment had no effect on plasma cholesterol levels (not shown). Moreover, GeneChip analysis of aortic arch mRNA samples from these mice (n = 4+5) showed a low number of differentially expressed genes and none of these were the 37 cholesterol-responsive genes.

### Regulatory Network Identification of Cholesterol Responsive Macrophage Genes

The rapid expansion of the lesions between weeks 30 and 40 leading to the formation of advanced plaques was primarily caused by lipid loading of foam cells already present at 30 weeks (clusters 1 and 3 in Figure 2A–2C). We suspected that some of the cholesterol-responsive genes identified at 30 weeks (Table S12) were essential to this process. To address this, total RNA was isolated from targeted and control THP-1 macrophages in cell culture that had been activated by PMA, treated with siRNA or mock and then incubated with acetylated-LDL (AcLDL) for 48 hours (see also Methods and Text S1). Expression data (Affymetrix Hu 130, 2.0+) was generated from 12 siRNA experiments and 4 pools of controls treated with unspecific siRNA (mock). Three transcripts, *GPR120* (Entrez Gene ID 338557), *GPR81* (Entrez Gene ID 27198) and *SOX6* (Entrez Gene ID 55553), were below the detection limits of the GeneChips suggesting that these genes were not active enough in this experimental model of foam cell formation to be detected or inactive. The remaining genes were organized in a 9-by-9 data matrix. Expression data for each gene was normalized by dividing with the mean expression level in controls followed by log-transformation. A linear gene regulation model
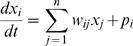
was fit to data as previously described [9]. Here *x* denotes expression data vectors, *W* is the network adjacency matrix, and *p* is the perturbation vector. In each knockdown experiment, the elements of *p* were −1 for the perturbed gene and 0 for all other genes. Note that because of the log-transform, this corresponds to a multiplicative model in actual expression levels. The algorithm controls the trade off between precision and recall by a single parameter δ. In our experiments we chose δ = 0.2. In simulations we found that this value corresponds to approx. 60% precision and 80% recall. Of note, interactions between genes (i.e. directed edges with stimulating or repressing effect) do not imply direct biological interactions but in most instances indirect (for instances mediated by proteins, metabolites or even intermediate genes (with low expression level such as transcription factors)).

**Figure 4:**
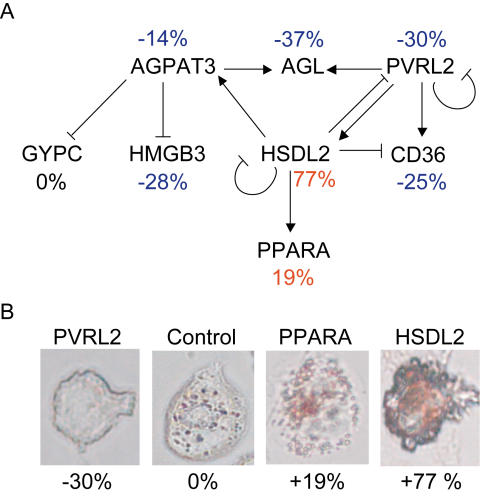
A regulatory gene network of foam-cell formation. Twelve cholesterol-responsive atherosclerosis genes (Table S12, in bold) were targeted in THP-1 macrophages using siRNA. Two days after transfection, siRNA-targeted macrophages and controls treated with nonspecific siRNA were incubated with AcLDL (50 µg/mL) for 48 hours; total RNA was isolated, and CE and lipid accumulation were determined. (A) Sixteen expression profiles (HG-U133_Plus_2 arrays, Affymetrix) from 12 siRNA experiments and four pooled controls were used to generate the regulatory gene network (Results) of 8 cholesterol-responsive genes involved in foam-cell formation, including PPARA and CD36. CE accumulation (given as average percentage next to each node) was decreased (blue) by siRNA inhibition of 5 genes and increased (red) by inhibition of 2 genes; inhibition of 1 gene had no effect (black) (see also Table 3). (B) Representative siRNA treated THP-1 cells after 48 incubation with AcLDL and stained with Oil-red-O.

*PRKAR2B* (Entrez Gene ID 5577) had no edges to the other nodes (e.g. genes). The remaining eight nodes were found to belong to a regulatory gene network (Figure 4A, Table 2). These nodes included *CD36* (Entrez Gene ID 948), which previously has been shown to promote foam-cell formation [10], and *PPARA* (Entrez Gene ID 5465) [11], which previously has been shown to prevent it [11]. Hydroxysteroid dehydrogenase-like 2 (*HSDL2*, Entrez Gene ID 84263) up-regulated *PPARA* and down-regulated *CD36*. Moreover, poliovirus receptor-related 2 (*PVRL2*, Entrez Gene ID 5819) also regulated *CD36*, increasing its expression and negatively regulating *HSDL2* and thus indirectly suppressing *PPARA* activity. From the regulation of *CD36* and *PPARA* in this regulatory network (Figure 4A), it can be predicted that inhibiting *PVRL2* would prevent foam-cell formation and inhibiting *HSDL2* would promote it. To validate the network, we tested these predictions by assessing the effect of inhibiting individual genes within the network on cholesterol-ester (CE) and lipid accumulation. The CE accumulations in the THP-1 cells in response to siRNA treatments were in agreement with our predictions (Figure 4A, Table 3). siRNA treatment against several of the other network nodes also affected CE accumulation in THP-1 macrophages (Figure 4A). Representative microscopic images of siRNA treated macrophages stained wit Oil-red-O are shown in Figure 4B.

**Table 2:** siRNA inhibition of eight cholesterol-responsive network genes in THP-1 macrophages incubated with acetylated LDL.

Gene symbol	Control (relative mRNA levels)	siRNA knock (relative mRNA levels)	*P*-value	siRNA inhibition (% relative control)	siRNA assay ID^a^
AGL	1.04±0.09	0.37±0.17	<0.001	64	110579
AGPAT3	1.00±0.09	0.31±0.04	<0.001	69	112079
CD36	1.02±0.12	0.35±0.02	<0.001	66	105939
GYPC	1.00±0.09	0.48±0.11	<0.001	52	11041
HSDL2	1.41±0.13	0.36±0.03	<0.001	74	33815
HMGB3	1.10±0.05	0.38±0.04	<0.001	65	145247
PPARA	1.19±0.20	0.37±0.05	<0.001	69	291496
PVRL2	1.26±0.16	0.44±0.03	<0.001	65	12251

a siRNA assays purchased from Ambion.

**Table 3:** Effects of siRNA inhibition of eight cholesterol-responsive network genes on cholesterol-ester accumulation in THP-1 macrophages incubated with acetylated LDL.

Gene symbol	Control (relative CE levels)	siRNA knock (relative CE levels)	*P*-value	CE content (% relative control)	Oil-Red-O staining
AGL	100±15	62±22	<0.001	−37	decreased
AGPAT3	98±17	85±17	ns	−14	decreased
CD36	89±2	67±2	<0.001	−25	decreased
GYPC	100±34	99±52	ns	nd	-
HSDL2	100±15	177±10	<0.001	+77	increased
HMGB3	97±10	70±20	ns	−28	decreased
PPARA	126±5	150±13	<0.05	+19	increased
PVRL2	100±8	70±13	0.002	−30	decreased

CE, cholesterol ester; nd, no difference; ns, not significant.

## Discussion

In this study, we first used genome-wide expression profiling to identify a point before which atherosclerosis developed slowly and only low-level inflammation was present. After this point, lesions expanded rapidly and inflammation increased markedly. The inflammation persisted in late phases, leading to the formation of advanced plaques, but lesion size increased transiently during a 10-week period. The rapid lesion expansion was primarily caused by an equally rapid lipid accumulation in macrophages. In early lesions, macrophages with low degree of inflammation and low lipid content accumulated. At a certain point, this accumulation halted and was followed by a rapid increase in macrophage lipid content paralleled by an induction of inflammatory genes. Lowering plasma cholesterol at this point prevented the rapid expansion of the lesions and the formation of advanced plaques. The cholesterol-lowering effect was mediated by 37 cholesterol-responsive atherosclerosis genes. Validation of the macrophage-related part of these genes by additional transcriptional profiling of mRNA from siRNA-targeted THP1─macrophages incubated with AcLDL exposed a regulatory gene network of foam-cell formation. This network as a whole and some of its individual nodes (particularly *PVRL2* and *HSDL2*) should be considered for therapies to prevent the formation of advanced plaques.

Transcriptional profiles of atherosclerosis lesions are challenging to interpret [3]. Such lesions contains several cell types, and the average mRNA contribution of a given cell type is altered with disease development. Thus, changes in mRNA levels represent a mixture of actual changes in cellular mRNA concentrations and changes in cell type admixture. In addition, cells in different stages of proliferation and differentiation (e.g., macrophages differentiation into foam cells) adds to this problem also within a given cell type. However, the current lesion mRNA dataset over tight time intervals provides, to our best knowledge, the most comprehensive compendium of lesion expression profiles representing known and novel biological processes and pathways activated during atherogenesis (Figure 2A).

Analysis of lesion mRNA clusters (Figure 2A) indicated that, at first, macrophages infiltrate the arterial wall, leading to the formation of fatty streaks. Then, at what appears to be a rather specific time point, these cells become activated, leading to a burst of inflammatory activity that, in combination with a rapid accumulation of CE in macrophages, generates advanced plaques; documented as a critical step in atherogenesis [12]. We believe this transformation can be related to the density of macrophages in the arterial wall. At a given density, the macrophages not only stimulate themselves (autocrine) but also stimulate each other (paracrine), leading to a burst of inflammatory activities and increasing lipid uptake. If such a mechanism is also present in humans, the timing of therapies to prevent or slow atherosclerosis development may be very important. Indeed, in these mice, the formation of advanced plaques was all together prevented by genetic lowering of plasma cholesterol immediately before the rapid lesion expansion (Figure 3A).

In contrast to mRNA profiles isolated during lesion development, the extent and relative composition of different cell types in the lesions were similar before and after the subacute lowering of plasma cholesterol (Figure 3B–3D). Thus, in this case the changes in mRNA concentration monitored by the GeneChips reflect actual changes in cellular mRNA levels. Since the accumulation of lipids in macrophages was a central process in the rapid lesion expansion and formation of advance plaques (clusters 1 and 3 in Figure 2A–2C), we used siRNA in THP-1 macrophages incubated with AcLDL to validate the cholesterol-responsive genes (Table S12). Eight genes were found to belong to a regulatory gene network in which *PVRL2* and *HSDL2* had central roles. Little is known about PVRL2 and HSDL2 (generating 6 and 3 hits in PubMed, respectively). A sequence variant in *PVRL2* is associated with the severity of multiple sclerosis [13]. *HSDL* encodes the sterol carrier protein-2, a small intracellular basic protein domain that enhances the transfer of lipids between membranes *in vitro* [14].

Although pointing out isolated candidates as being more promising than others is tempting, the lesson from whole-genome approaches will most certainly be that complex diseases like atherosclerosis will not be defeated by targeting single genes (probably not even single regulatory genes) but instead to target several genes belonging to a similar network of pathology [9],[15]. This notion is supported by studying a small gene network as the present (Figure 4A) where none of the nodes (i.e., genes) can be said to solely promote or inhibit foam-cell formation.

Our findings imply that the timing of interventions with plasma cholesterol-lowering agents may be very critical. Patients at risk of developing complications of atherosclerosis (e.g., stroke and MI) may benefit from being treated very early in life. The development of noninvasive technologies to detect early atherosclerosis or molecular markers of atherosclerosis stages will be important in this respect. For normo-cholesterolemic individuals but with other atherosclerosis risk factors, novel regimens targeting atherosclerosis genes that mediate the beneficial effects of plasma cholesterol-lowering may be useful. In summary, the network of cholesterol-responsive atherosclerosis target genes and regulators of foam-cell formation identified in our study merits further attention.

## Materials and Methods

### The Mouse Model

The *Ldlr* ^−/−^ *Apob* ^100/100^ *Mttp* ^flox/flox^Mx1-*Cre* mouse model has a plasma lipoprotein profile similar to that of familial hypercholesterolemia, which causes rapid progression of atherosclerosis [7]. For *Mttp* deletion, mice were injected with 500 µl of pI-pC (1 µg/µl; Sigma, St. Louis, MO) every other day for 6 days to induce *Cre* expression, thereby recombining *Mttp* (*Mttp* ^Δ/Δ^) or not in the *Ldlr* ^−/−^ *Apob^100/100^Mttp* ^wt/wt^ Mx1-*Cre* mice. Littermate controls received PBS (*Mttp* ^flox/flox^). The study mice had been back crossed 5 times to C57BL/6 (<5% 129/SvJae and >95% C57BL/6), were housed in a pathogen-free barrier facility (12-hour light/12-hour dark cycle), and were fed rodent chow containing 4% fat. Genotypes were determined by polymerase chain reaction (PCR) with genomic DNA from tail biopsies. Plasma cholesterol and triglyceride concentrations were determined in non-fasted blood samples with colorimetric assays (Infinity cholesterol/triglyceride kits; Thermo Electron, Melbourne, Australia), and plasma glucose levels with Precision Xtra (MediScience, Cherry Hill, NJ).

### *En Face* Analysis and Histology

Aortas were pinned out flat on black wax surfaces as described [16], stained with Sudan IV, photographed with a Nikon SMZ1000 microscope, and analyzed with Easy Image Analysis 2000 software (Tekno Optik, Skärholmen, Sweden). Lesion area was calculated as a percentage of the entire aortic surface between the aortic root and the iliac bifurcation. Aortic roots were isolated and immediately frozen in liquid nitrogen in OCT compound (Histolab, Västra Frölunda, Sweden). Cryosections (20 µm) were cut and stained with hematoxylin (Htx) and Oil-red-O as described [17]; other sections (6–8 µm) were incubated first with rat anti-mouse CD68 antibody or a control antibody (Serotec, Oxford, England) overnight at 4°C and then with fluorescent anti-rat IgG (Vector Laboratories, Burlingame, CA) and counterstained with mounting medium containing DAPI (Vector Laboratories).

### Transcriptional Profiling

Aortas were perfused with RNAlater (Qiagen, Valencia, CA), and the aortic arch from above the third rib to the aortic root was removed and homogenized with FastPrep (Qbiogene, Irvine, CA). Total RNA was isolated with RNeasy Mini Kit (Qiagen) using a DNAse I treatment step. RNA quality was assessed with a Bioanalyzer 2100 (Agilent Technologies, Santa Clara, CA). High-quality RNA samples (32 from *Mttp* ^flox/flox^, 5 from *Mttp* ^Δ/Δ^, and 9 *Mttp* ^wt/wt^ mice) were used for global gene expression measurements with cDNA arrays (Mouse Genome 430 2.0 GeneChips, Affymetrix, Santa Clara, CA) at 10 (n = 7, PBS), 20 (n = 5, PBS), 30 (n = 6, PBS+5, pI-pC), 40 (n = 5), 50 (n = 5), and 60 (n = 4) weeks. In addition at 30 weeks, 9 *Mttp* ^wt/wt^ (n = 5, PBS+4, pI-pC). All samples were prepared with the two-cycle protocol recommended by the manufacturer. Arrays were scanned with GeneChip Scanner 3000 and analyzed with GeneChip Operating Software (Affymetrix).

### Text Mining and Prior Atherosclerosis Knowledge

Automated text mining of PubMed was used to establish lists of genes related to atherosclerosis, foam cells, smooth muscle cells, endothelial cells, and T cells. Briefly, a gene was considered related if it co-occurred with any of the following terms in the abstract of an article in PubMed: *atherosclerosis*, *arteriosclerosis* (“atherosclerosis-related”, Table S1), *foam cell*, *macrophage*, *monocyte* (“foam cell related”, Table S2), *smooth muscle cell* (Table S3), *endothelial cell* (Table S4), and *T-cell* (Table S5). These tables constitute fairly comprehensive but not specific lists of genes with possible roles in atherosclerosis or in the cell types involved in atherosclerosis (i.e., may contain false positives but a low number of false negatives) with a substantial overlap (Figure S1). We also generated a list of “established” atherosclerosis genes by manually extracting from recent reviews genes known to be important in atherosclerosis (Table S6).

### siRNA of THP-1 Macrophages Incubated with Acetylated-LDL

Monocytes of the human monocytic cell line THP-1 were plated in six-well culture dishes (Becton Dickinson Labware, NJ) at 6 × 10^5^ cells/well in 10% fetal calf serum (FCS)-RPMI-1640 medium with L-glutamine (2 mM) and HEPES buffer (25 mM) (Gibco-Invitrogen, Carlsbad, CA) supplemented with penicillin (100 U/mL) and streptomycin (100 µg/mL) (PEST) and induced to differentiate into macrophages with phorbol 12-myristate 13-acetate (PMA) (50 ng/mL) (Sigma) for 72 hours. For each gene, cells were transfected with up to three siRNAs (Ambion, Austin, TX), using Lipofectamine 2000 according to the manufacturer's instructions (Invitrogen), in medium without FCS, PEST, and PMA. Two days after transfection, siRNA-targeted macrophages and mock-treated controls (nonspecific siRNA) were incubated with AcLDL (50 µg/mL) for 48 hours in 1% FCS medium with PEST. AcLDL was prepared as described [18]. The samples were dialyzed against PBS at 4°C. AcLDL protein concentration was determined by the Bradford method. LDL was isolated from the plasma of healthy donors by sequential ultracentrifugation [19].

### Lipid, Protein, and Gene Expression Measurements

For lipid imaging, THP-1-derived foam cells were fixed with 10% formaldehyde in PBS for 10 min and washed twice with PBS. The cells were stained with Oil-red-O (0.3% in 60% isopropanol) for 20 min, washed twice with 60% isopropanol and twice with PBS, and examined with a Nikon Eclipse E800 microscope at 40× magnification. Lipids were isolated by hexan/isopropanol (3∶2) extraction at room temperature for 1 hour followed by 0.5 ml chloroform for 15 min [20]. The lipid extracts were dried and resuspended in 80 µl of isopropanol with 1% Triton-X-100 (Sigma). The lipid content of the foam cells was determined by enzymatic assays using the Infinity kit for total cholesterol (Thermo Electron) and a kit for free cholesterol (Wako Chemicals, Richmond, VA). After lipid extraction, proteins were extracted from the same wells by incubation with 0.5 M sodium hydroxide for 5 hours at 37°C. Protein concentration was determined by the Bradford method.

For HG-U133_Plus_2 array analysis (Affymetrix) and to determine the degree of knockdown by siRNA, total RNA was isolated from the AcLDL-incubated THP-1 cells with RNeasy Mini-kit (Qiagen). The concentration was determined with a spectrophotometer (ND-100, NanoDrop Technologies, Wilmington, DE). For cDNA synthesis, 0.5 µg of total RNA was reverse transcribed with Superscript II (Invitrogen) according to the manufacturer's protocol. After 5-fold dilution, cDNA (3 µL) was amplified by real-time PCR with 1×TaqMan universal PCR master mix (Applied Biosystems, Foster City, CA) on an ABI Prism 7000 (PE Biosystems) and software according to the manufacturer's protocol. Assay-On-Demand Kits containing corresponding primers and probes from Applied Biosystems were used, and expression values were normalized to acidic ribosomal phosphoprotein P0. Each sample was analyzed in duplicate (Text S1).

### Statistics and Calculations

Differences in the mRNA levels of selected genes, mouse plasma measurements, and lesion surface areas between time points were analyzed with unpaired *t* tests. Gene expression signal-level data were computed with MAS 5.0 (Affymetrix) using default settings, log-transformed, and normalized to total intensities (global scaling). After normalization, signal intensities were computed for each gene in the Mouse Genome Informatics Database (MGD genes, Jackson Laboratory, www.jax.org) by averaging the signal of the corresponding Affymetrix probe sets. Of the 11,979 GeneChip probe sets (Mouse Genome 430 2.0 GeneChips, Affymetrix) that had no match in the database, 1.5% were differentially expressed (false discover rate (FDR) <0.05, n = 177), representing the fraction of genes/probe sets that were not considered for further analyses. The remaining 33,122 probe sets had at least one match in 19,879 MGD genes (of a total of 32,095). Lowess normalization [21] was applied in pair-wise fashion before differential expression testing. To correct for multiple testing when computing probabilities of differential expression and FDRs, we used empirical Bayes statistics [8]. Clustering was performed with the FindCluster algorithm in Mathematica 5.1 (Wolfram Research, Champaign, IL). GO and pathway analyses were performed with EASE software [22]. The regulatory gene network of THP-1 macrophages incubated with AcLDL was inferred as described [9].

## Supporting Information

Figure S1 Relations between the cell-type textmining lists in Tables S2, S3, S4, and S5
(0.36 MB EPS)

Table S1 Textmining list, Atherosclerosis
(0.24 MB XLS)

Table S2 Textmining list, Macrophages
(0.32 MB XLS)

Table S3 Textmining list, Smooth muscle cells (SMCs).
(0.30 MB XLS)

Table S4 Textmining list, Endothelial cells
(0.22 MB XLS)

Table S5 Textmining list, T-cells
(0.37 MB XLS)

Table S6 List of established atherosclerosis genes with t-test p-values
(0.03 MB XLS)

Table S7 Cell-specific markers.
(0.02 MB XLS)

Table S8 Gene list, lesion cluster 1
(0.06 MB XLS)

Table S9 Gene list, lesion cluster 2
(0.06 MB XLS)

Table S10 Gene list, lesion cluster 3
(0.06 MB XLS)

Table S11 Gene list, lesion cluster 4
(0.05 MB XLS)

Table S12 List of 37 cholesterol-responsive genes.
(0.03 MB XLS)

Table S13 GO analysis, lesion cluster 1
(0.21 MB XLS)

Table S14 GO analysis, lesion cluster 2
(0.15 MB XLS)

Table S15 GO analysis, lesion cluster 3
(0.17 MB XLS)

Table S16 GO analysis, lesion cluster 4
(0.13 MB XLS)

Table S17 KEGG and Biocarta pathway analysis, lesion cluster 1
(0.01 MB XLS)

Table S18 KEGG and Biocarta pathway analysis, lesion cluster 2
(0.01 MB XLS)

Table S19 KEGG and Biocarta pathway analysis, lesion cluster 3
(0.01 MB XLS)

Table S20 KEGG and Biocarta pathway analysis, lesion cluster 4
(0.01 MB XLS)

Table S21 KEGG and Biocarta pathway analysis, all genes
(0.01 MB XLS)

Table S22 Transcription factors in lesion cluster 1
(0.02 MB XLS)

Table S23 Transcription factors in lesion cluster 2
(0.01 MB XLS)

Table S24 Transcription factors in lesion cluster 3
(0.02 MB XLS)

Table S25 Transcription factors in lesion cluster 4
(0.01 MB XLS)

Text S1 Supporting Methods
(0.03 MB DOC)
