# Supplementary material for: Transcriptional Profiling Uncovers a Network of Cholesterol-Responsive Atherosclerosis Target Genes
Source: PLoS Genet. 2008 Mar 14;4(3):e1000036. doi: 10.1371/journal.pgen.1000036 (PMC2265530; doi:10.1371/journal.pgen.1000036)
Supplement: Text S1 — Supporting Methods (0.03 MB DOC) [file pgen.1000036.s027.doc]

**Supporting Methods-Skogsberg et al.**

Cholesterol-responsive atherosclerosis genes identification

Cholesterol-responsive atherosclerosis gene were considered those genes that were differently expressed (FDR<0.05) in the atherosclerotic aortic arch of mice in which *Mttp* recombination in the liver (see also Methods) had been induced by intra peritoneal injections with 500 μl pIpC (1 μg/ μl) compared to PBS injected controls (see Table S5, n =37). The injections were performed at four sequential time points with two days interval starting at the first day of week 29 continuing until the end of week 29. pIpC-treatment achieved a lowering of plasma cholesterol with 80% or more in all mice as measured in plasma at sacrifice at 30 weeks. Plasma cholesterol levels in control mice treated with saline were unaffected. During week 30, the mice were left alone to wash out any remaining effects of the injections.

Cholesterol-responsive genes selected for siRNA targeting

Of the 37 identified cholesterol-responsive genes, 27 had preidentified Taqman and siRNA assays (Applied Biosystems and Ambion, respectively). In Table S5, 12 of these 27 genes are marked in bold indicating that they previously have been reported as expressed by THP-1 macrophages. These were targeted by silencing interfering RNA (siRNA). Among these genes were *CD36* [1] and *PPARA* [2] with previously shown involvement in atherosclerosis.

**References**

1. Nicholson AC, Han J, Febbraio M, Silversterin RL, Hajjar DP (2001) Role of CD36, the macrophage class B scavenger receptor, in atherosclerosis. Ann N Y Acad Sci 947: 224-228.

2. Hennuyer N, Tailleux A, Torpier G, Mezdour H, Fruchart JC, et al. (2005) PPARalpha, but not PPARgamma, activators decrease macrophage-laden atherosclerotic lesions in a nondiabetic mouse model of mixed dyslipidemia. Arterioscler Thromb Vasc Biol 25: 1897-1902.
